# Reconstructing the Post‐Burn Auricle: A Microsurgical Approach Using a Contralateral Free Temporoparietal Fascial Flap and Autologous Costal Cartilage Framework

**DOI:** 10.1002/ccr3.72852

**Published:** 2026-06-12

**Authors:** Siming Wei, Yongjun Chen, Liwei Dong

**Affiliations:** ^1^ Department of Plastic and Reconstructive Surgery Xijing Hospital of Fourth Military Medical University Xi'an China

**Keywords:** auricular burn, auricular reconstruction, costal cartilage framework, free temporoparietal fascial flap, microsurgery

## Abstract

Post‐burn auricular defects compromise both aesthetics and facial symmetry, often imposing significant social and psychological burdens that severely diminish patients' quality of life. Scarring in the mastoid region following burns typically exhibits poor elasticity and texture, along with compromised cutaneous blood supply. Scar contracture further leads to displacement and deformation. These factors collectively contribute to the considerable complexity and variability inherent in post‐burn auricular reconstruction. The intricate three‐dimensional structure of the auricle itself renders its restoration a formidable challenge in plastic and reconstructive surgery. In this study, we present a single‐stage reconstruction strategy for post‐burn ear deformity applicable when local skin is unsuitable and the ipsilateral temporoparietal fascia is non‐viable. The technique involves framework fabrication using autologous costal cartilage, followed by coverage with a contralateral free temporoparietal fascial flap. We propose that this approach offers a valuable and effective option for auricular reconstruction in such complex scenarios.

## Key Clinical Message

For patients with severe post‐burn ear deformities and local scarring, single‐stage auricular reconstruction using an autologous costal cartilage framework covered by a contralateral free temporoparietal fascial flap is a sound, effective surgical strategy.

## 1 Introduction

The prominent and exposed position of the auricle renders it susceptible to injury, making it a common site of involvement in craniofacial trauma. Auricular defects arise from various etiologies, with burns representing one of the most frequent causes [1]. Such defects not only compromise aesthetics but also impair practical functions, such as wearing masks or eyeglasses. Furthermore, they disrupt facial symmetry, often leading to significant psychosocial distress and a marked reduction in quality of life. The intricate three‐dimensional structure of the auricle makes its post‐burn reconstruction a formidable challenge in plastic and reconstructive surgery.

Successful ear reconstruction requires consideration of both the cartilage framework and skin coverage, while satisfactory skin coverage is imperative in achieving the desired shape of the reconstructed ear. Therefore, an adequate volume of thin, pliable soft tissue in the preauricular region is considered ideal. Typically, local flaps—such as the ipsilateral temporoparietal fascial flap, mastoid skin flap, or expanded flaps from the mastoid or retroauricular area—serve as the primary options. Post‐burn deformities, however, present additional complexity due to extensive scarring, damage to adjacent soft tissues, and a paucity of healthy local skin, rendering ipsilateral reconstruction especially difficult. In this study, we describe a single‐stage reconstruction technique for post‐burn auricular deformity. The method involves fabricating a framework from autologous costal cartilage, which is then covered using a contralateral free temporoparietal fascial flap. We propose that this approach represents a valuable and viable surgical strategy when local soft tissue is insufficient or unsuitable for reconstruction.

## 2 Case History

### 2.1 Patient

A 22‐year‐old male patient presented with burn sequelae manifesting as right temporal bone exposure, nuchal scarring, and right auricular defect. Initial surgical management involved a series of procedures, including scalp flap transposition for coverage of the exposed temporal bone and split‐thickness skin grafting for the reconstruction of burn wounds in the right temporal and nuchal regions. Eighteen months after the initial injury, the patient presented to our institution for right auricular reconstruction. Written informed consent from the patient was obtained according to journal guidelines.

**FIGURE 1:**
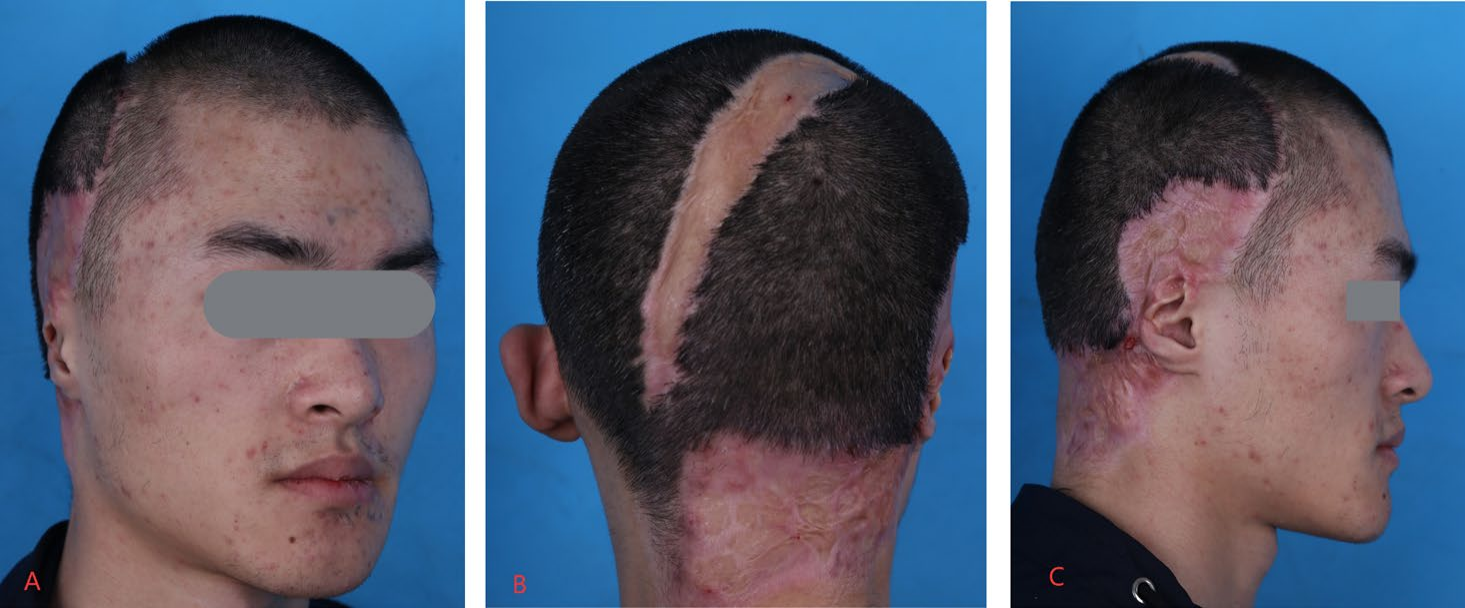
(a–c) Preoperative photographs demonstrating a post‐burn defect involving the superior auricle, with conspicuous scarring in the mastoid and temporal regions.

**FIGURE 2:**
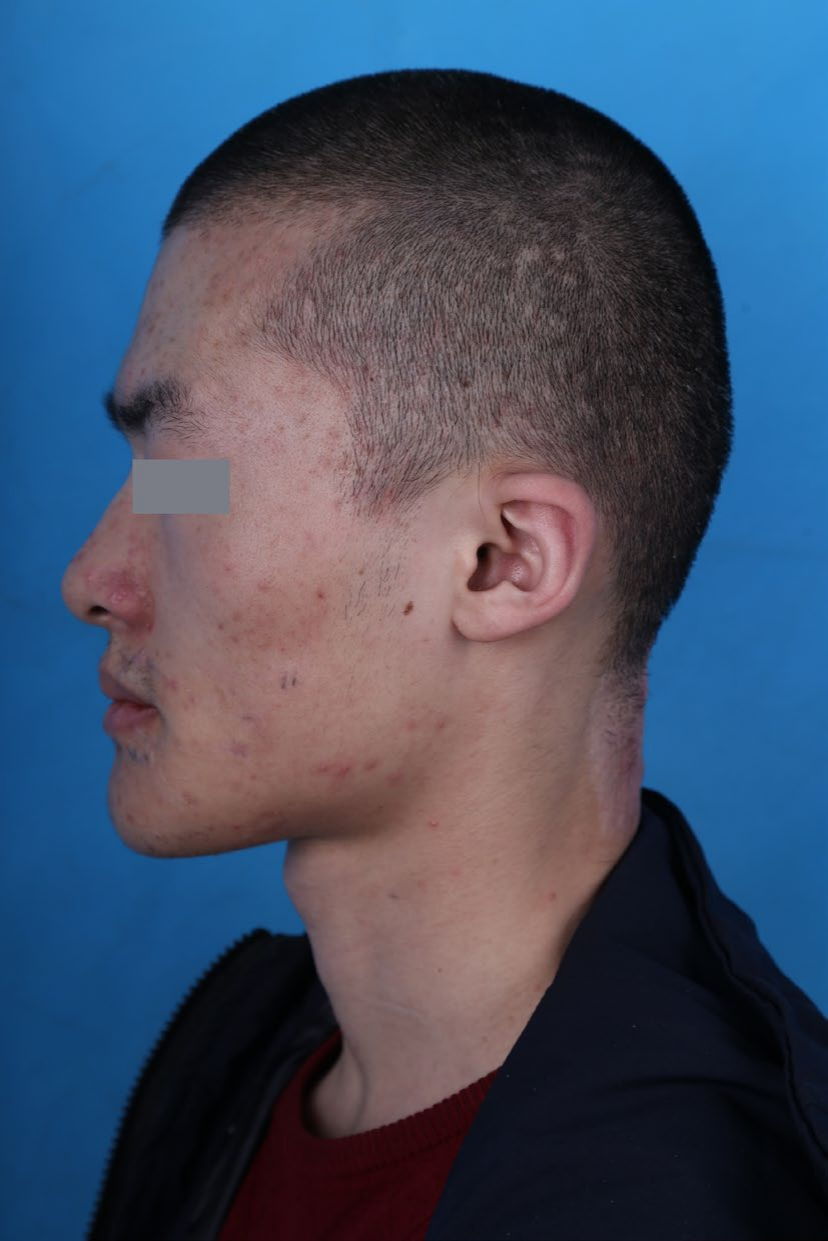
Preoperative lateral photograph of the contralateral healthy ear.

A preoperative physical examination revealed the absence of the right auricle, with an area of hypertrophic scarring measuring 8 cm × 5 cm present in the right temporal region. A near‐complete destruction of the normal three‐dimensional architecture of the right auricle was noted. The helix was largely absent, with only a small, scarred remnant of the lower helix remaining that lacked natural curvature and continuity. The antihelix, scapha, and triangular fossa were completely obliterated and devoid of identifiable anatomical landmarks, having been replaced by dense, inelastic scar tissue. The concha was partially preserved but severely distorted, exhibiting irregular borders and reduced depth due to surrounding scar contracture. The lobule was present but demonstrated severe superior retraction, resulting in a loss of its normal pendulous shape and volume. The tragus showed partial residual tissue but was displaced and distorted with indistinct borders, while the antitragus was completely unidentifiable. The scalp flap transfer site is visible on the head, with the donor area from the vertex to the occipital region showing the appearance of skin graft repair. Additionally, scarring alopecia is present in the nape of the neck. Extensive scarring in the right temporal region resulted in compromised tissue availability for conventional auricular reconstruction. A preoperative ultrasound examination of the right superficial temporal vessels revealed occlusion of the vessels beneath the temporal scar. Consequently, a single‐stage auricular reconstruction was planned utilizing an autologous costal cartilage framework in conjunction with a free contralateral temporoparietal fascial flap (Figure 1a–c). Prior to the ear reconstruction surgery, a scalp pre‐expansion procedure was performed to address scarring on the head and right temporal region. Alopecia resulting from scarring was repaired by the expanded scalp flap. The contralateral auricle is healthy and free from injury (Figure 2).

### 2.2 Flap Design and Surgical Technique

Following the induction of general anesthesia, the patient was positioned supine. The surgical fields—encompassing the head, neck, face, and chest—were prepared using standard aseptic technique, which included scrubbing with iodophor solution and draping with sterile sheets. The procedure was conducted by two surgical teams operating concurrently. Team A harvested the right costal cartilage graft and obtained a split‐thickness skin graft from the scalp. Team B dissected the left temporoparietal fascial flap, carved the costal cartilage into an auricular framework, and subsequently performed the framework implantation and auricular sculpting.

Team A: An incision line was marked overlying the right costal margin. A 4‐cm longitudinal skin incision was made parallel to the 7th rib, adjacent to the sternum. Meticulous dissection was carried down through the tissue layers to expose the rib surface. The perichondrium was incised and carefully elevated from the underlying cartilage using a periosteal elevator, taking care to protect the underlying pleura. Segments of the 7th and 8th ribs were then resected. The harvested cartilage was immediately wrapped in saline‐soaked gauze and set aside. Hemostasis within the donor site was meticulously achieved. The perichondrial layer and overlying muscular tissues were reapproximated in layers using 5–0 absorbable sutures. The subcutaneous tissue was closed with 4–0 absorbable sutures. The skin edges were precisely approximated. External reinforcement was provided using adhesive tension strips, and the site was finally dressed with a sterile, occlusive bandage. Next, tumescent local anesthesia was administered to the right parietal scalp. A split‐thickness skin graft, measuring approximately 12 cm by 4 cm, was harvested from this site. The donor wound was covered with petrolatum‐impregnated gauze and sterile dressings, followed by the application of a pressure dressing to secure it.

**FIGURE 3:**
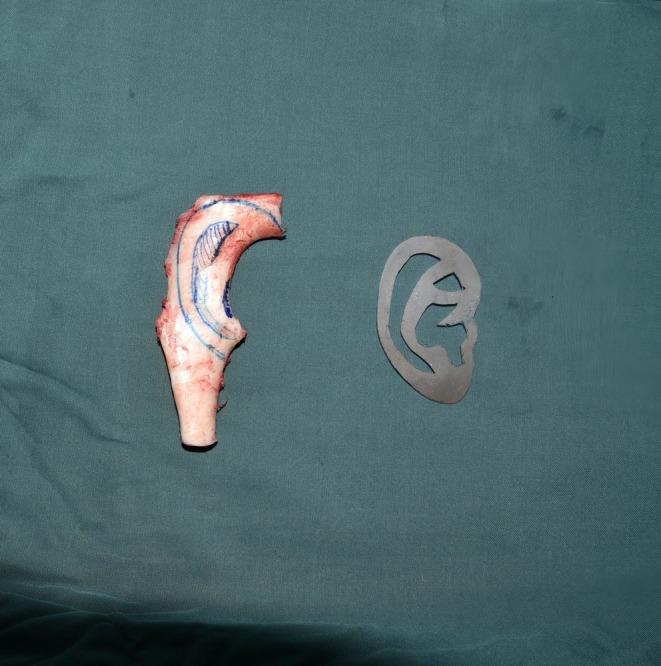
Schematic overlay of the contralateral (healthy) auricle used as a template for sculpting the costal cartilage framework.

**FIGURE 4:**
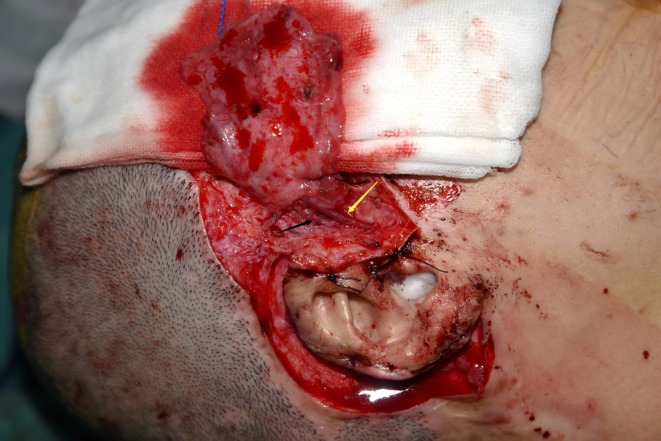
Intraoperative view following microsurgical transfer of the contralateral temporoparietal fascial flap to the recipient site, demonstrating well‐perfused tissue. The yellow color indicates the artery after anastomosis, while the red arrow indicates the vein.

**FIGURE 5:**
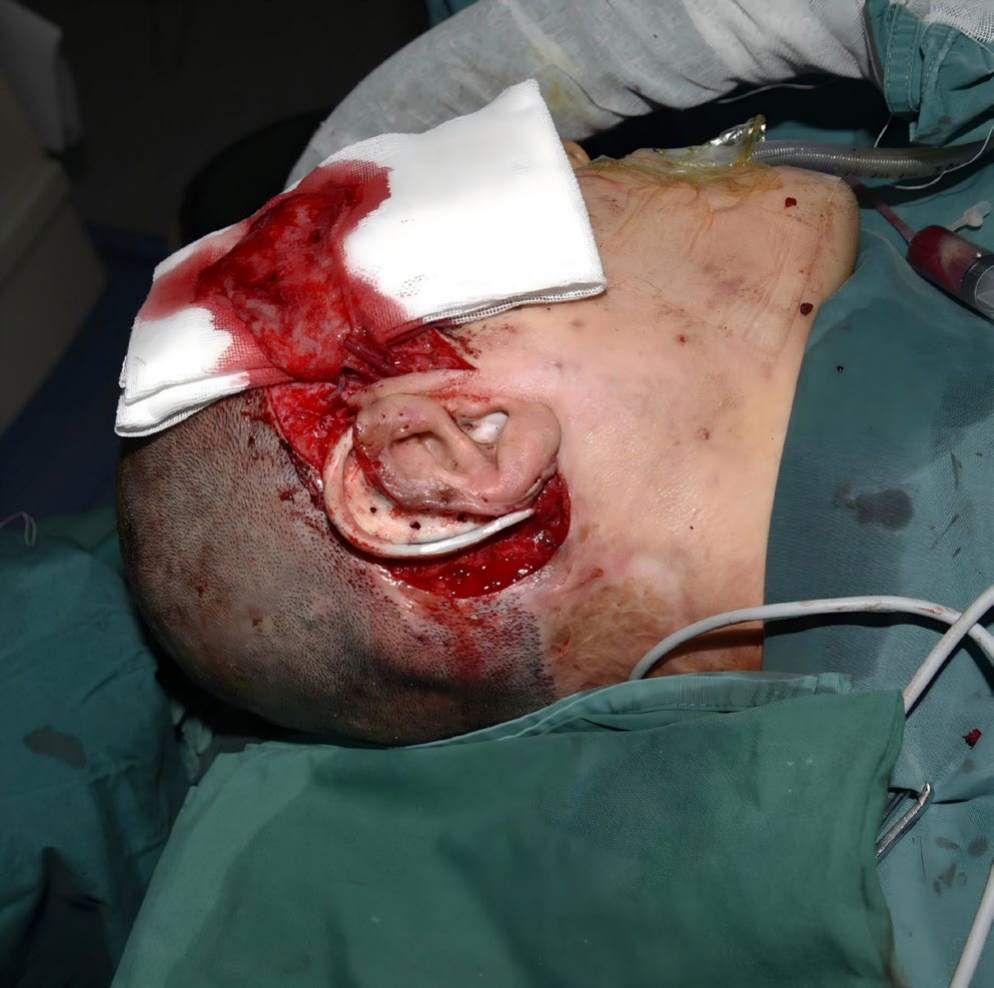
Placement of the carved costal cartilage framework into the auricular defect.

Team B: Using the healthy contralateral auricle as a template, the antihelix was carved from the costal cartilage and secured with stainless steel wire sutures to fabricate the framework (Figure 3). The framework was then immersed in normal saline until use. An incision was made along the line of adhesion between the right auricular remnant and the scalp scar, extending through the skin and subcutaneous tissue down to the fascial plane. The residual auricular tissue was elevated, and any hyperplastic scar tissue posterior to the remnant was meticulously debrided. Hemostasis was achieved using a combination of moist gauze pressure and bipolar electrocautery. A left temporoparietal fascial flap was subsequently elevated. A Y‐shaped incision was designed over the temporoparietal region. Subcutaneous infiltration with lidocaine was performed along the marked incision and within the planned flap boundaries. The scalp was incised down to the level of the superficial temporal fascia, and a scalp flap was raised. A superficial temporal fascial flap, incorporating the superficial temporal artery and vein, was dissected in an inverted trapezoidal shape, measuring approximately 11 cm × 5 cm with a pedicle width of 4 cm. Meticulous dissection was performed to preserve the integrity of the superficial temporal vessels and the scalp hair follicles. Hemostasis was secured with direct pressure and electrocautery. A single drain was placed, and the scalp incision was closed in layers. The vascular pedicle of the harvested temporoparietal fascial flap was dissected to an adequate length. The flap was then stored in heparinized sterile normal saline. In the right auricular region, the superficial temporal artery and vein anterior to the planned recipient site were meticulously isolated and mobilized to provide sufficient length. The free fascial flap was transferred to the retroauricular recipient site on the right side and secured with peripheral sutures. End‐to‐end microvascular anastomoses were performed using 11–0 monofilament nylon sutures: the flap vein to the recipient superficial temporal vein, and the flap artery to the recipient superficial temporal artery. Papaverine (30 mg) was administered topically around the arterial anastomosis. Following release of the tourniquet, successful flap perfusion was confirmed immediately (Figure 4 and Video 1). The temporoparietal fascial flap was then wrapped around the cartilage framework and sutured in place using 5–0 absorbable sutures to create a natural cranioauricular angle (Figure 5).

**VIDEO 1:** Upon completion of vascular anastomosis during auricular reconstruction, the contralateral temporoparietal fascial flap exhibited immediate robust arterial pulsation and adequate tissue perfusion, with a total ischemia time of 15 s, thereby confirming successful revascularization. Video content can be viewed at https://onlinelibrary.wiley.com/doi/10.1002/ccr3.72852.

**FIGURE 6:**
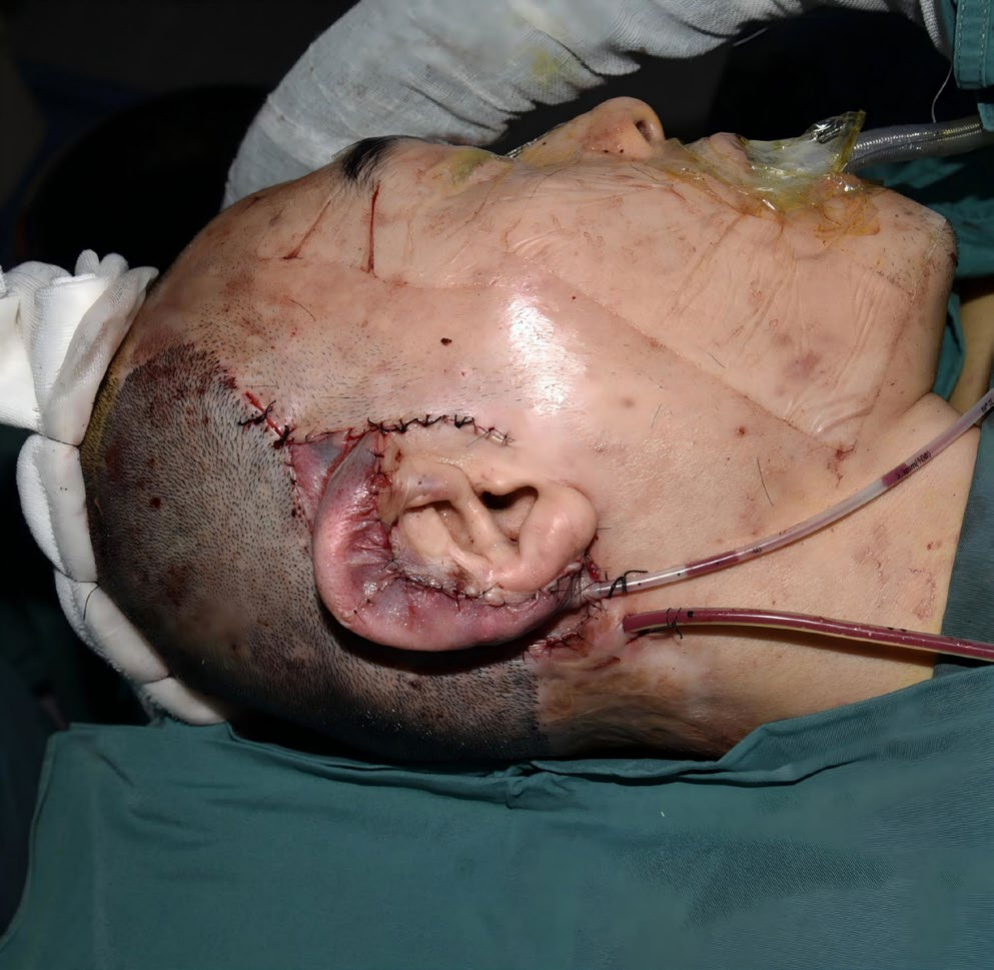
Immediate postoperative appearance of the reconstructed auricle.

Two closed‐suction drains were placed: one between the cartilage framework and the overlying fascia, and another in the subcutaneous plane beneath the scalp. The cutaneous defect over the reconstructed auricle was covered with the pre‐harvested split‐thickness scalp skin graft, which was secured using 6–0 non‐absorbable sutures. A standard sterile dressing was applied. Postoperatively, the patient received a course of intravenous antibiotics for infection prophylaxis. The drains were removed when the 24‐h output was less than 1 mL (Figure 6). Skin sutures were removed between postoperative days 10 and 12. The reconstructed auricle was protected from pressure, and the patient was scheduled for regular outpatient follow‐up.

## 3 Result

In the two‐week postoperative follow‐up, the vascular anastomosis remained patent, the surgical site healed primarily, and skin grafting on the temporal fascia demonstrated excellent survival rates. No complications such as infection or flap necrosis were observed. When compared with conventional auricular reconstruction techniques, the use of microsurgical temporal fascia free flap transplantation results in more pronounced swelling of the reconstructed ear in the short term postoperatively. At the eight‐month postoperative follow‐up, the reconstructed auricle had significantly reduced swelling and exhibited a relatively natural shape, although the outcome was not as ideal as that achieved with conventional ear reconstruction surgery. The color and texture of the skin grafts on the fascia surface demonstrated minimal variation when compared to normal skin. No significant hypertrophic scarring was observed at the junction between the reconstructed auricle and the native auricle.

**FIGURE 7:**
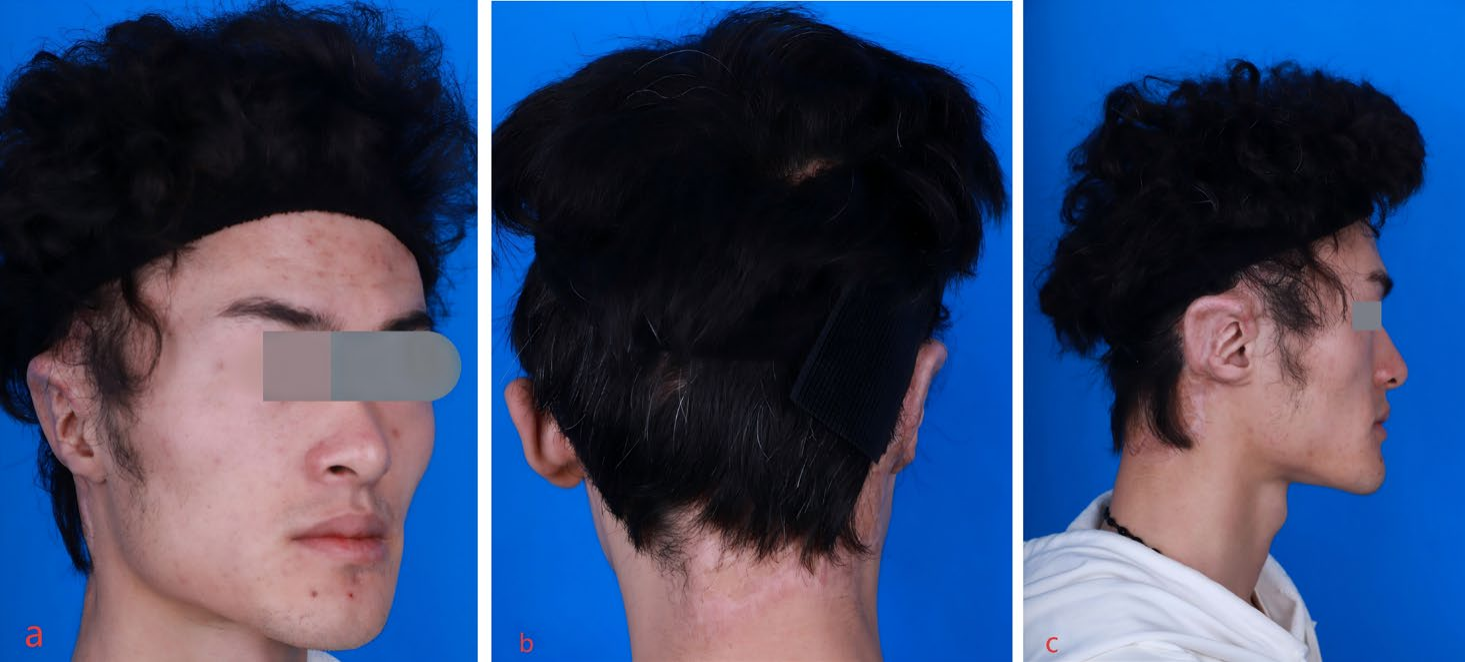
(a–c) Postoperative photographs at 6‐month follow‐up showing the final contour and integration of the reconstructed auricle.

Based on the recovery of morphological characteristics of the reconstructed auricle, the author had suggested that the patient undergo a revision procedure to debulk the auricular flap for further improvement of the auricle's appearance. Nevertheless, the patient considered the surgical outcome to be acceptable and therefore declined the author's recommendation for a second operation. No long‐term donor‐site complications, such as thoracic deformity or severe hypertrophic scarring at the recipient site, were observed. Functionally, the patient reported no impairment in wearing eyeglasses or facial masks (Figure 7a–c).

## 4 Discussion

Auricular reconstruction under conventional conditions is a well‐established surgical technique with generally favorable postoperative outcomes. However, reconstruction following partial burn‐related defects entails unique clinical considerations. Auricular reconstruction following partial burn‐related defects entails unique clinical considerations. The presentation of residual auricular tissue and the characteristics of post‐burn scarring vary significantly across patients. Of particular concern are the mastoid region scars, which typically exhibit poor elasticity and unfavorable texture, accompanied by compromised dermal perfusion. Scar contracture frequently leads to local tissue distortion and malposition, collectively contributing to the technical complexity and procedural variability inherent in post‐burn auricular reconstruction. These factors not only elevate the difficulty of surgical repair but also present a substantial clinical challenge in plastic and reconstructive surgery. The primary reconstructive strategy depends on the quality of periauricular scar tissue and the availability of adjacent soft tissue. Local flaps are generally considered the first‐line option when conditions permit. In the present case, however, significant scarring and exposed bone in the right temporal region raised concerns regarding the viability of an ipsilateral temporoparietal fascial flap. Moreover, the use of a local flap carried a high risk of inadequate tissue volume for complete defect coverage. Consequently, when conventional auricular reconstruction techniques are no longer viable, microsurgical approaches offer a valuable alternative. Among these, the free temporoparietal fascial flap, first reported by Park, has demonstrated favorable outcomes based on existing literature. In contrast, the use of a free forearm flap is more controversial due to the sacrifice of a major forearm vessel and the associated risk of impaired hand function [2, 3, 4]. Notably, despite the initial success reported by Park in total auricular reconstruction using a contralateral free temporoparietal fascial flap with autologous costal cartilage, publications on this specific technique have been notably scarce over the following two decades.

The temporoparietal fascia flap (TPFF) is widely utilized in the field of reconstructive repair in plastic surgery, predominantly for the reconstruction of soft tissue defects in the head and neck regions as well as the distal extremities. Owing to its inherent flexibility and thinness, the TPFF serves as an ideal option for soft tissue defect coverage. Furthermore, the denervation of the TPFF leads to progressive thinning in the late postoperative period, which further enhances its applicability in reconstructive procedures. In the management of soft tissue defects secondary to hand trauma, TPFF coverage over the joint surface can achieve favorable functional and aesthetic outcomes for the affected hand [5]. Additionally, the TPFF can improve facial symmetry in the treatment of unilateral facial paralysis [6]. Its application in the field of nerve repair also merits attention: in the treatment of recurrent nerve adhesion of the superficial branch of the radial nerve, the TPFF is used as a nerve wrapping material, which effectively prevents recurrent nerve adhesion and thereby significantly ameliorates the clinical symptoms of patients [7]. Moreover, the TPFF has demonstrated promising efficacy in the management of cerebrospinal fluid fistulas following skull base and posterior cranial fossa surgeries by obliterating dead space and covering defective areas [8].

The selection of the free TPFF for auricular reconstruction in the present case was primarily based on its following distinct advantages. First, the application of the TPFF in auricular reconstruction can effectively address the issue of blood supply: its abundant vascularity ensures a robust blood circulation when used to cover the auricular reconstruction framework, which in turn reduces the risk of infection and promotes wound healing [9]. Second, the flexibility and thinness of the TPFF render it an optimal choice for wrapping the auricular reconstruction framework, which can effectively decrease the incidence of postoperative complications [10]. Third, the use of the free TPFF for auricular reconstruction is associated with reduced postoperative scar formation and high levels of patient satisfaction [11, 12]. Finally, the free TPFF can serve as a viable alternative when the pedicled temporoparietal fascia flap is unavailable due to various contraindications, providing effective soft tissue coverage and support for the surgical defect [13].

### 4.1 Harvesting an Adequate Temporoparietal Fascia Flap

The TPFF is predominantly supplied by the superficial temporal artery and vein. Its vascular anatomy is favorable for microsurgical transfer, featuring a pedicle with consistent location, large‐caliber vessels, and reliable perfusion, which collectively contribute to high flap survival rates. The vessels arborize extensively within the fascia, creating a rich vascular network [14]. The flap can be safely harvested in dimensions up to 12–14 cm by 14–17 cm, with a typical thickness of 2–4 mm [15, 16], providing ample tissue for coverage. For ear reconstruction, a flap of sufficient size is critical. It must be large enough to completely envelop the three‐dimensional cartilage framework while allowing for the creation of a well‐defined auriculocephalic sulcus. It is noteworthy that the anterosuperior aspect of the TPFF is naturally thinner than the posteroinferior portion. The use of a split‐thickness skin graft from the scalp, as employed in this case, offers several distinct advantages. It provides an excellent color and texture match to the periauricular skin, demonstrates high graft take rates, and is associated with minimal postoperative contracture. Furthermore, the scalp donor site heals with minimal scarring and without alopecia, as the hair follicles remain intact in the deeper dermal layers.

### 4.2 Sculpting the Costal Cartilage Framework

Auricular reconstruction can employ various materials for the underlying framework, including auricular cartilage, costal cartilage, porous polyethylene, titanium mesh, and silicone implants. Autologous cartilage is generally considered the graft material of choice. As native tissue, it offers the inherent advantages of excellent biocompatibility, elimination of rejection risk, and permanent integration into the host. Costal cartilage, typically harvested from the 6th to 9th ribs, possesses distinct structural properties that make it particularly suitable for this purpose. It provides sufficient volume, favorable elasticity, and intrinsic shape memory, allowing it to be carved into a stable, three‐dimensional framework capable of replicating the complex auricular topography. Furthermore, it demonstrates long‐term resilience against mechanical stress and maintains its structural integrity.

### 4.3 Postoperative Care and Protection

Defined postoperative contouring is essential for optimal aesthetic outcomes. For a period of 1 month, gentle but specific compression is applied to the recessed areas of the reconstructed auricle, such as the triangular fossa, scaphoid fossa, and concha, using molded cotton bolsters. Concurrently, a custom‐made protective splint is worn to shield the ear from inadvertent pressure during the early healing phase, thereby preventing loss of projection or framework deformation. In the event of postoperative venous congestion, prompt management is critical to mitigate elevated perfusion pressure and preserve flap viability. Established interventions include needle decompression, medicinal leech therapy, and the application of chemical leech (hirudin‐based) agents.

The surgical procedure is also associated with several technical challenges. When utilizing expanded flaps for auricular reconstruction, strict attention must be paid to hemostasis and negative pressure application to ensure prompt and intimate adherence between the flap and the cartilage framework. In contrast, for a free fascial flap, minor bleeding near the main vascular pedicle often requires a more conservative approach to hemostasis. Furthermore, due to systemic heparinization during microvascular anastomosis, the flap itself tends to exhibit increased postoperative oozing. Negative pressure drainage must also be applied judiciously; both the placement of drains and the degree of suction must be carefully managed to avoid compromising flap perfusion. Postoperatively, hematoma formation between the fascial flap and the cartilage framework remains a potential complication, which can lead to poor adherence and compromise the final aesthetic outcome.

## 5 Conclusion

In summary, the free temporoparietal fascial flap (FTPFF) provides a robust and pliable soft tissue envelope for auricular reconstruction. For patients with post‐burn ear deformities characterized by severe local scarring and compromised viability of the ipsilateral temporoparietal fascia, the described single‐stage technique—employing an autologous costal cartilage framework covered by a contralateral FTPFF—represents a sound and effective surgical strategy. This approach not only addresses the unique challenges of such complex defects but also enriches the reconstructive armamentarium available to the plastic surgeon.

## Author Contributions

**Siming Wei:** data curation, formal analysis, writing – original draft. **Yongjun Chen:** formal analysis. **Liwei Dong:** data curation, project administration, resources.

## Funding

The authors have nothing to report.

## Conflicts of Interest

The authors declare no conflicts of interest.

## Data Availability

The data that support the findings of this study are available in the Supporting Information of this article and/or from the corresponding author upon reasonable request, subject to patient confidentiality obligations and institutional ethical approval. All relevant clinical details are presented within the manuscript itself. Due to the nature of the case and privacy/ethical restrictions, individual‐level patient data cannot be made publicly available.
